# Protective effect of hydrogen-rich saline against radiation-induced immune dysfunction

**DOI:** 10.1111/jcmm.12245

**Published:** 2014-03-12

**Authors:** Sanhu Zhao, Yanyong Yang, Wen Liu, Zhiqiang Xuan, Shouming Wu, Shunfei Yu, Ke Mei, Yijuan Huang, Pei Zhang, Jianming Cai, Jin Ni, Yaoxian Zhao

**Affiliations:** a Zhejiang Provincial Center for Disease Control and PreventionZhejiang, China; b Department of Radiation Medicine, Second Military Medical UniversityShanghai, China; c Department of Radiology, Shanghai First People*s HospitalShanghai, China

**Keywords:** ionizing radiation, hydrogen, radioprotection, apoptosis, immune regulation

## Abstract

Recent studies showed that hydrogen can be used as an effective radioprotective agent through scavenging free radicals. This study was undertaken to evaluate the radioprotective effects of hydrogen on immune system in mice. H_2_ was dissolved in physiological saline using an apparatus produced by our department. Spleen index and histological analysis were used to evaluate the splenic structural damage. Spleen superoxide dismutase, GSH, MDA were measured to appraise the antioxidant capacity and a DCF assay for the measurement of radical oxygen species. Cell apoptosis was evaluated by an Annexin V-FITC and propidium iodide staining method as well as the apoptotic proteins such as Bcl-2, Bax, caspase-3 and c-caspase-3. CD4+ and CD8+ T cells subtypes were detected by flow cytometry with FITC-labelled antimouse CD4 and PE antimouse CD8 staining. Real-time PCR was utilized to determine the CD4+ T cell subtypes and related cytokines. Our study demonstrated that pre-treatment with H_2_ could increase the spleen index and attenuate the radiation damage on splenic structure. Radical oxygen species level was also reduced by H_2_ treatment. H_2_ also inhibited radiation-induced apoptosis in splenocytes and down-regulated pro-apoptotic proteins in living mice. Radiation-induced imbalance of T cells was attenuated by H_2_. Finally, we found that H_2_ could regulate the polarization of CD4+ T cells and the level of related cytokines. This study suggests H_2_ as an effective radioprotective agent on immune system by scavenging reactive oxygen species.

## Introduction

The immune system is one of the most important defence mechanisms against various environmental agents including ionizing radiation. Thus, epidemiological long-term studies demonstrated that ionizing radiation could induce a dose-dependent impairment of the immune response as well as a persistent inflammatory status with deregulation of cytokines production [1,2]. Furthermore, the dysregulation of immune homeostasis, in particular the function of CD4+ T cells, may influence health status and impede tissue repair after exposed to ionizing radiation [3]. Dainiak *et al*. [4] stated that ionizing radiation could result in a massive killing of blood cells such as lymphocytes and even in a halting of the proliferation of haematopoietic progenitors, thereby leading to suppression of immune function, and increasing the risk of infection while impairing wound healing. Besides immunosuppression, ionizing radiation also can induce the pro-inflammatory processes, in which tumour necrosis factor (TNF)-α/interferon (IFN)-γ and interleukin (IL)-4 levels were altered and finally lead to the disorder of inflammatory [5].

Early in 1975, Dole *et al*. found that hydrogen (H_2_) can induce a marked regression of the skin squamous cell carcinoma by acting as a free radical decay catalyser [6]. In 2007, Ohsawa *et al*. reported that H_2_ could selectively reduce hydroxyl radical [7]. Since then, H_2_ has become to the forefront of therapeutic medical gas research. Our department also found that H_2_ could protect cultured cells and mice from ionizing radiation, and exerted protective effects on the gastrointestinal tract, haematology and spermatogenic epithelium [8,9]. Meantime, our research also showed that hydrogen can reduce the apoptosis of splenocytes caused by radiation [10]. In this study, we found that hydrogen treatment could reduce radiation damage on spleen and reverse the radiation-induced imbalance of T cells, which suggest H_2_ as an effective modulator on the immune dysfunction.

## Materials and methods

### Preparation of hydrogen-rich saline

H_2_ was dissolved in physiological saline for 6 hrs under high pressure (0.4 MPa) to a supersaturated level using a hydrogen-rich water-producing apparatus produced by our department. The saturated H_2_ saline was stored under atmospheric pressure at 4°C in an aluminium bag with no dead volume. Hydrogen-rich saline was freshly prepared every week, which ensured that a concentration of more than 0.6 mmol/l was maintained. Gas chromatography was used to confirm the content of H_2_ in saline by the method described by Ohsawa *et al*. [7].

### Irradiation

^60^Co-gamma rays in the irradiation centre (Faculty of Naval Medicine, Second Military Medical University, China) were used for irradiation. Mice (with or without H_2_ pre-treatment) were exposed to different doses of radiation, depending on the requirements of this study.

### Animals and experimental design

All the protocols were approved by the Second Military Medical University, China in accordance with the Guide for Care and Use of Laboratory Animals published by the US NIH (publication No. 96-01). Male C57BL/6 mice (8 weeks old, weighing 22 ± 1 g) were used in the experiments. The animals were housed in individual cages in a temperature-controlled room with a 12 hrs light/dark cycle, and food and water were provided *ad libitum*.

Animals were divided into three groups as follows: (1) the Normal group: male C57BL/6 mice remained untreated; (2) the single radiation group: male C57BL/6 mice were treated intraperitoneally (IP) with physiological saline (5 ml/kg) 5 min. before radiation; and (3) the H_2_ group: male C57BL/6 mice were treated IP with hydrogen-rich saline (5 ml/kg) 5 min. before radiation. For the experiment, both radiation group and H_2_ group were irradiated with 5 Gy. And eight mice per group were used in this study.

### Spleen index and histology

At 14 days after irradiation, mice were killed by cervical dislocation under isoflurane anaesthesia, and the weights of the body and the spleen were recorded. Spleen specimens were dehydrated, embedded in paraffin and then sectioned into slices with the thickness of 5 μm. After then they were dewaxed in xylene and rehydrated by exposure to graded ethanols, tissue sections were stained with Haematoxylin–Eosin for light microscopy.

### Biochemical assays

At 24 hrs after exposure, spleen was removed for biochemical assays. Spleen superoxide dismutase (SOD) activity was determined by using the SOD assay kit-WST (Dojindo, Kumamoto, Japan) based on the inhibition of the formation of NADHPMS-NBT complex. The glutathione (GSH) was measured using total glutathione quantification kit (Dojindo) based on the development of a yellow colour when 5′,5′-dithiobis 2-nitrobenzoic acid was added to compounds containing sulphydryl groups. According to the manufacturer*s instructions, the levels of malondialdehyde (MDA) were assessed by a MDA assay kits (Cell BioLabs, San Diego, CA, USA).

### Western blotting analysis

The protein concentration was determined with bovine serum albumin as a standard by a Bradford assay. Equal amounts of protein preparation (10 μg in 10 μl buffer) were analysed on SDS-polyacrylamide gels, electro-transferred to nitrocellulose membranes and blotted with a primary antibody against bcl-2 (1:1000; Cell Signaling, Boston, MA, USA), bax (1:1000; Cell Signaling), caspase-3 (1:1000; Cell Signaling), c-caspase-3 (1:1000; Cell Signaling), tubulin (1:1000; Cell Signaling). Incubation with the primary antibody was conducted overnight at 4°. Incubation with peroxidase-conjugated anti-rabbit secondary antibody (1:10,000; Cell Signaling) was performed for 120 min. at room temperature. Bound antibodies were then visualized using a chemiluminescence reaction and Hyperfilm ECL. For densitometrical quantification of the autoradiographies, the Multi Analyst software was applied.

### Flow cytometry analysis

Mice were killed at 24 hrs after irradiation, after which spleen were immediately removed. Cells were dispersed by passage through a fine wire mesh into a 35 × 10 mm petri dish containing 1 ml PBS. Isolated splenocytes were washed three times with PBS, and then stained with Annexin V-FITC and propidium iodide by an Apoptosis Detection Kit (Invitrogen, Carlsbad, CA, USA), according to the manufacturer*s instructions.

Lymphocyte suspensions obtained from mice were washed and resuspended in 200 μl flow cytometry buffer (PBS solution supplemented with 3% foetal calf serum). Cell-surface molecules were immunostained for 30 min. at 37° in the dark with directly labelled antibodies against CD4 and CD8 (FITC antimouse CD4 and PE antimouse CD8; Biolegend, San Diego, CA, USA). Cells were analysed by flow cytometry (Beckman Coulter, Pasadena, CA, USA).

### Real-time quantitative polymerase chain reaction

Total RNA was extracted from the spleen samples with the RNeasy Mini Spin Kit (TAKARA, Dalian, China), and cDNA was prepared with the SuperScript RT Reagent Kit (TAKARA). SYBR chemistry (TAKARA) was used to amplify PCR; primers were designed with Primer Express software (Applied Biosystems, Foster City, CA, USA) and listed in Table 1. The PCR was performed with the Roche Lightcycler 2.0 (Basel, Switzerland).

**Table 1:** Oligonucleotides used in the real-time PCR

	Sequence (5′ to 3′)
GAPDH	AGCAGTCCCGTACACTGGCAAAC
	TCTGTGGTGATGTAAATGTCCTCT
T-bet	CCCCTTCTCACCTCTTCTATCC
	TCCGCTTCATAACTGTGTTCC
GATA-3	TGAAGAAAGAAGGCATCCAGA
	AGTGGCTGAAGGGAGAGATGT
RORγt	TTCAGTATGTGGAGTTTGC
	AAAAAGACTGTGTTGTTGG
Foxp3	CTTTCACCTATGCCACCCTTA
	CATCTACGGTCCACACTGCTC
IFN-γ	TACTGCCACGGCACAGTCATTGAA
	GCAGCGACTCCTTTTCCGCTTCCT
IL-12	TGTCAATCACGCTACCTCCTC
	CAGGTCTTCAATGTGCTGGTT
IL-4	GTCATCCTGCTCTTCTTTCTCG
	CTCTCTGTGGTGTTCTTCGTTG
IL-5	GATGAGGCTTCCTGTCCCTAC
	CTCGCCACACTTCTCTTTTTG
IL-10	TACAGCCGGGAAGACAATAACT
	CCTGCATTAAGGAGTCGGTTAG
IL-17	TATCCCTCTGTGATCTGGGAAG
	ATCTTCTCGACCCTGAAAGTGA
IL-23	GGGAACAAGATGCTGGATTG
	ACTGGATACGGGGCACATTA
TGF-β1	AACAATTCCTGGCGTTACCTT
	GAATCGAAAGCCCTGTATTCC
TNF-α	CCTATGTCTCAGCCTCTTCTCAT
	CACTTGGTGGTTTGCTACGA
IL-1β	TGAAATGCCACCTTTTGACAG
	CCACAGCCACAATGAGTGATAC
GM-CSF	CATCAAAGAAGCCCTGAACC
	CGCATAGGTGGTAACTTGTGTT

### Detection of ROS *in vivo*

The procedure for the detection of radical oxygen species (ROS) in the spleen is followed as the method described by Laggner *et al*. [11]. DCFH (10 μl, 1:1000) was injected in anesthetized mice spleen. Thirty minutes after irradiation, spleen was quickly dissected from the anesthetized mice and prepared for cryosection. Fluorescence images were acquired by using a fluorescent microscope. The acquired images were analysed by semiquantitative comparisons of the relative fluorescence intensity of testes between groups.

### Statistical analysis

We used one-way anova followed by Scheffe*s test to analyse any difference between control and irradiated cultures at each single culture time-point. All reported values are expressed as means ± SEM for each experiment. The number of samples is indicated in the description of each experiment. *P* < 0.05 was considered statistically significant.

## Results

### Morphological changes of spleen

**Fig. 1:**
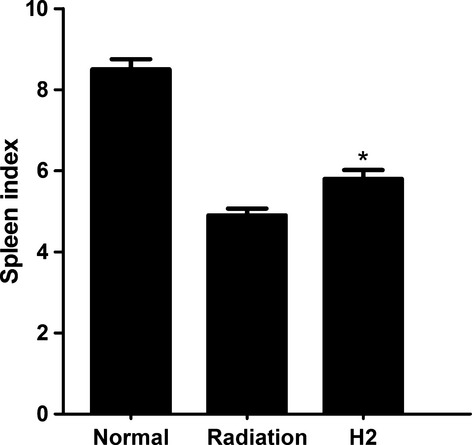
Spleen index 14 days after irradiation. Spleen index = spleen weight/bodyweight × 100. The data were expressed as means ± SEM (*n* = 8), *P < 0.05.

At 14 days post irradiation, mice were killed and the spleen index was calculated (spleen index = spleen weight/bodyweight × 100). The index of spleen in the H_2_ group was significantly higher than that of the single radiation group (Fig. 1).

**Fig. 2:**
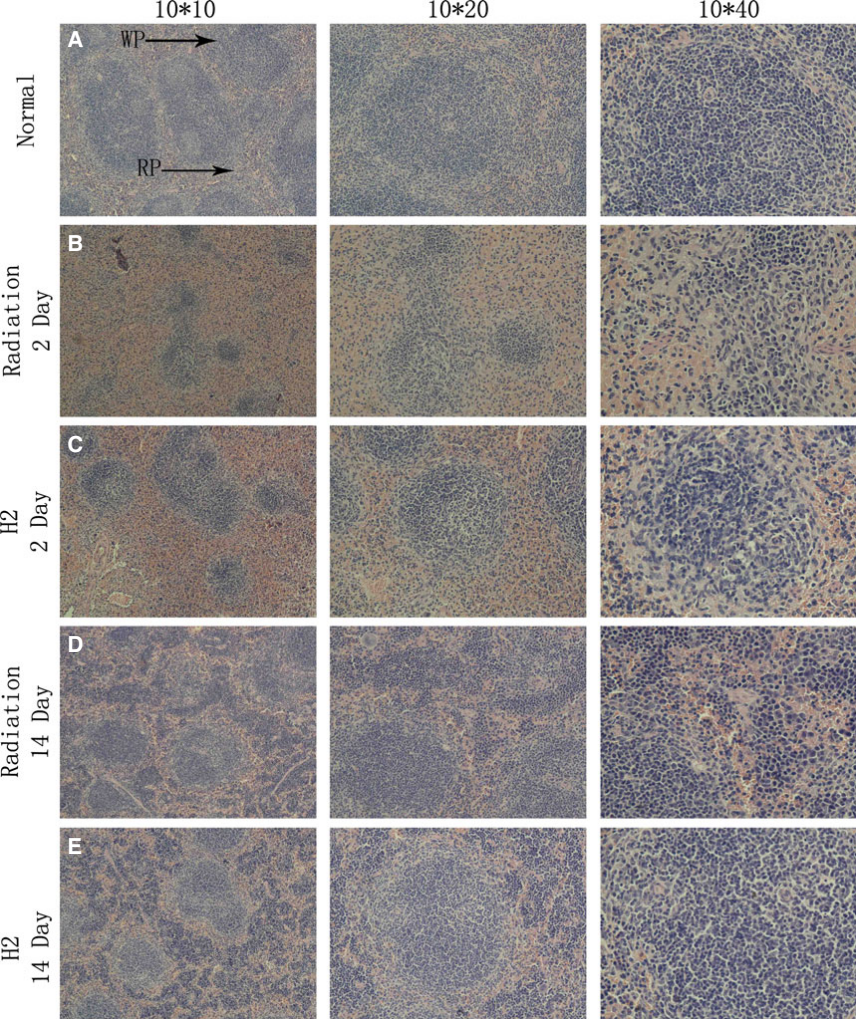
Morphology of spleen after *in vivo* γ-radiation. (**A**) Spleens of mice with control morphology are comprised of both red and white pulps. (**B**) Spleens of mice 2 days after *in vivo* γ-radiation. (**C**) Spleens in mice pre-treated with H2 at 2 days after irradiation. (**D**) Spleens of mice 14 days after *in vivo* γ-radiation. (**E**) Spleens in mice pre-treated with H2 at 14 days after irradiation.

The splenic morphology was characterized by using the following subjective descriptors: control, diminished, depleted when compared with normal group (Table 2). Cellularity of white pulp decreased significantly after exposure to 5 Gy irradiation. Both width and density of the layer of lymphocytes appeared to decrease obviously. Cellularity of red pulp was obviously reduced and accompanied by tissue congestion. And the resulting morphology was described as depleted (Fig. 2B). Compared with Figure 2B, the reduction in cellularity of white pulp and red pulp was less remarkable and the morphology was characterized as diminished (Fig. 2C). Fourteen days post exposure, cellularity of both white pulp and red pulp in radiation group and H_2_ group recovered, while the structure in radiation group was much disorganized and the density of cells were lower compared with the H_2_ group (Fig. 2D and E).

**Table 2:** Morphological descriptors for histological sections of mice spleen after *in vivo* exposure to γ-radiation

Control	Indistinguishable from unexposed animals
Diminished	Decreased cellularity of red and white pulps when compared with control morphology
Depleted	Decreased cellularity of red and white pulps when compared with diminished morphology

### Changes of oxidative stress–related parameters

**Fig. 3:**
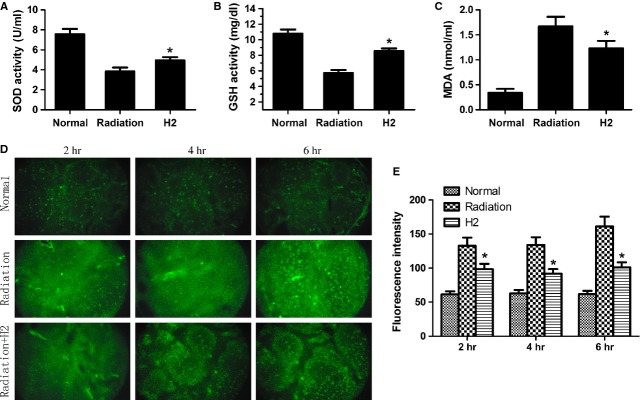
Effects of hydrogen to antioxidant indexes. Changes in (**A**) levels of spleen superoxide dismutase and (**B**) activities of GSH, and (**C**) concentration of MDA in normal, radiation and H2-pre-treated mice. (**D**) DCF-DA fluorescence images were obtained at 2, 4, 6 hours after 5 Gy radiation. (**F**) DCFH-DA fluorescence intensity was semiquantified from the cryosection of each independent experiment. The data were expressed as means ± SEM (*n* = 8), *P < 0.05.

The levels of enzymatic antioxidants (SOD) and the activities of non-enzymatic antioxidant (GSH) in each group are shown in Figure 3A. H_2_ restored SOD and GSH, and the results indicated that H_2_ pre-treatment seemed to restore spleen antioxidant status. As shown in Figure 3B, cellular MDA concentration at 24 hrs after radiation in the H_2_ group was significantly lower than that of the radiation group.

We also detected the ROS level by a DCFH assay. As shown in Figure 3C and D, ROS levels were much lower in the H_2_ group than that in the radiation group.

### Anti-apoptosis effects of hydrogen

**Fig. 4:**
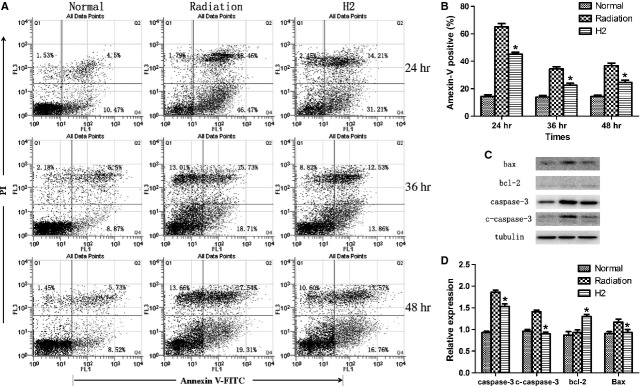
Hydrogen attenuated irradiation-induced splenocytes apoptosis. (**A** and **B**) Irradiation-induced splenocytes apoptosis rate was quantified by Flow Cytometry at 24, 36, 48 hrs after exposure. (**C**) Bcl-2, Bax, caspase-3, c-caspase-3 protein expression was detected by western blot at 14 days after exposure. (**D**) Normalization of Bcl-2, Bax, caspase-3, c-caspase-3 expression to Tubulin. The data were expressed as means ± SEM (*n* = 8), *P < 0.05.

To detect the anti-apoptosis effects of hydrogen against radiation, flow cytometry and western blotting analysis were used at different times after the last radiation. As shown in Figure 4A and B, after whole body irradiation, apoptosis of splenocytes was significantly enhanced, while the percentage of apoptotic cells decreased significantly in H_2_ group.

To investigate the possible mechanism whereby H_2_ attenuates radiation-induced apoptosis, caspase-3, c-caspase-3, bax and bcl-2 level were examined by western blot analysis. The results showed that caspase-3/c-caspase-3 and bax expression markedly decreased in H_2_-pre-treated, while bcl-2 expression was increased by H_2_ (Fig. 4C and D).

### Effects of hydrogen on radiation-induced disorder in T cell

**Fig. 5:**
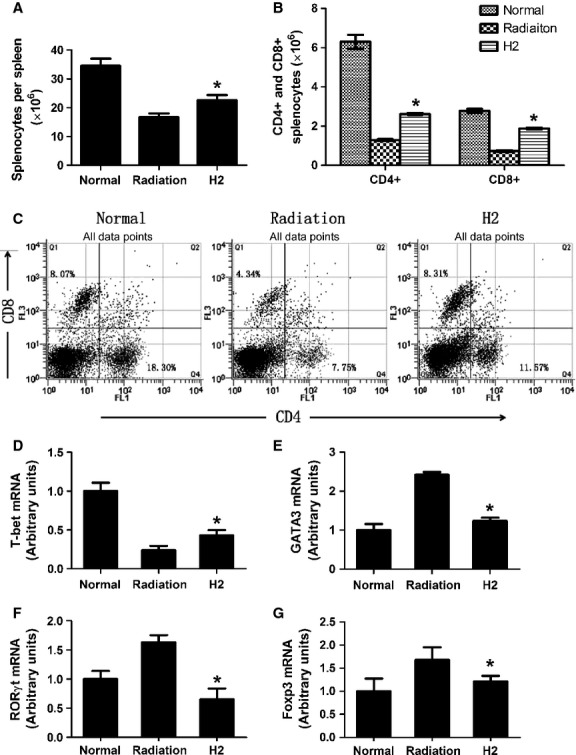
Effects of hydrogen on radiation-induced disorder in T cell. (**A**) The total number of splenocytes in normal, radiation, H2-pre-treated mice 14 days after exposure. (**B** and **C**) The absolute number and percentage of CD4 + and CD8 + T cells in normal, radiation, H2-pre-treated mice 14 days after exposure. (**D**–**G**) T-bet, GATA-3, Foxp-3, RORγt mRNA expression was detected by real-time PCR and normalized to GAPDH. The data were expressed as means ± SEM (*n* = 8), *P < 0.05.

To investigate the effects of hydrogen on radiation-induced immune disorder, the total number of splenocytes and the per cent of CD4+ and CD8+ were measured. As shown in Figure 5A, the number of splenocytes decreased significantly in radiation group compared with that in H_2_ group. Both the absolute number and the percentage of CD4+ and CD8+ in radiation group were lower than those in H_2_ group (Fig. 5B and C).

CD4+ cells can be divided into Th1, Th2, Treg and Th17. All of them play important roles in immune system. Our results showed that hydrogen can down-regulate radiation-induced up-regulation of Th2, Th17 and Treg, and elevated radiation-induced decrease in Th1 (Fig. 5D–G).

### Cytokine levels in spleen

**Fig. 6:**
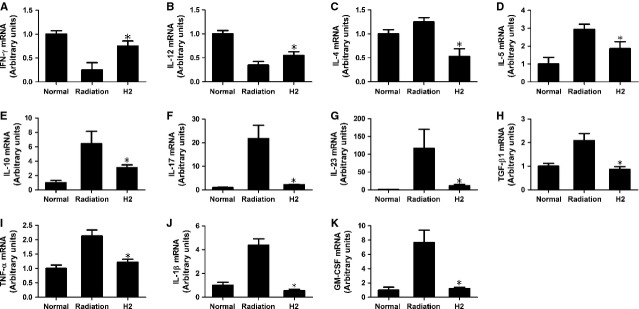
Effects of hydrogen on mRNA expression of Th-type cytokines and pro-inflammatory cytokines *in vivo*. (**A** and **B**) Effects of hydrogen on mRNA expression of Th1-type cytokines *in vivo*. (**C**–**E**) Effects of hydrogen on mRNA expression of Th2-type cytokines *in vivo*. (**F** and **G**) Effects of hydrogen on mRNA expression of Th17-type cytokines *in vivo*. (**H**) Effects of hydrogen on mRNA expression of Treg-type cytokines *in vivo*. (**I**–**K**) Effects of hydrogen on mRNA expression of pro-inflammatory cytokines *in vivo*. All those data were normalized to GAPDH. The data were expressed as means ± SEM (*n* = 8), *P < 0.05.

Real-time PCR was used to detect the levels of cytokine in spleen. Nine cytokines out of the eleven [TNF-α, transforming growth factor (TGF)-β1, GM-CSF, IL-1β, IL-4, IL-5, IL-10, IL-17, IL-23] measured in the spleen were elevated significantly in radiation group compared with normal group, while the levels in H_2_ group decreased compared with the single radiation group. Both IFN-γ and IL-12 decreased in radiation group and recovered to normal levels nearly in the H_2_ group (Fig. 6A–K).

## Discussion

The immune system is made up of a complex and vital network of cells and organs, which include bone marrow, thymus, spleen and lymph nodes, *etc* [12,13]. The immune dysfunction can result in autoimmune diseases (autoimmune hepatitis, autoimmune aplastic anaemia, autoimmune urticarial, *etc*.), inflammatory diseases and even cancer [14–16]. As one of the most important immune organs, the immunological function of spleen is mainly carried out by white pulp, which consists of aggregates of lymphoid tissue [17]. The injury of spleen can cause dysfunction of immune system and make one susceptible to infections and other diseases.

Recent research showed that the size and weight of spleen, as well as the amount of the white pulp and red pulp, was greatly reduced in irradiated mice [18]. The spleen index is an indicator of extent or status of the injury of spleen. In this study, the radiation protective effect of hydrogen on spleen was observed. In radiation group, the spleen index is 4.9 ± 0.12, whereas the spleen index is 5.8 ± 0.24 in H_2_ group, which indicated that hydrogen can rescue the decrease in spleen index caused by radiation, thus supporting the radioprotection effect on spleen.

White pulp and red pulp, which could be readily identified morphologically, can be as significant components to evaluate the functional state of the spleen. Because of the extreme sensitivity of lymphocytes to radiation, white pulp and red pulp will disappear quickly after exposure [19]. In this study, the structure of the spleen has been determined. The white pulp was destroyed seriously and the red pulp cells were also reduced, with surrounding hyperaemia in radiation group in both 2 and 14 days after exposure. While the tissue structures were effectively protected by hydrogen-rich solution.

Spleen superoxide dismutase, GSH and MDA are important in detecting protection from radiation [20]. In our study, the levels of SOD and GSH decreased significantly and MDA increased in irradiated mice. However, pre-treatment of hydrogen prior to radiation exposure increased the whole antioxidant activity. As most of ionizing radiation-induced damage is caused by ROS, we speculated that the ROS scavenging effect of hydrogen might play a vital role in radioprotection. In our study, DCFH, which has been successfully used to detect the level of ROS *in vitro* and *in vivo,* was used to investigate the radioprotective effect of H_2_. Our results showed that H_2_ pre-treatment could reduce the fluorescence intensity of the oxidized DCF significantly, preserve endogenous antioxidants and decrease spleen oxidative damage during IR. These results showed that hydrogen can act as a scavenger of free radicals and thereby exert important radioprotective effects.

Cell apoptosis induced by radiation is regulated by a complex balance between pro-apoptotic factors caspase-3, c-caspase-3 and Bax, as well as anti-apoptotic factors, the Bcl-2 family [21,22]. Previously, we reported that hydrogen could reduce the apoptosis of splenocytes and thymocytes caused by irradiation at 24 hrs post irradiation. And our present study showed that hydrogen could inhibit the apoptosis at even 36 and 48 hrs post irradiation. Meantime, we also found that hydrogen reduced radiation-induced caspase-3 and Bax activation, and enhanced bcl-2 level. Our results presented the evidence that hydrogen could block radiosensitive cells from entry into apoptosis, thus decreasing the damage to spleen.

It had been proved that T lymphocytes are extremely sensitive to ionizing radiation and radiosensitivity varies among different T cell subpopulations. In our study, we found that hydrogen reduced the decrease in total number of splenocytes per spleen and rescued the absolutely number and percentage of CD4+ and CD8+ T lymphocytes, thus protecting the immune system.

CD4+ T lymphocytes can be divided into four groups: Th1, Th2, Th17 and Treg cells. The balance among Th1, Th2, Th17 and Treg cells plays important roles in the development/prevention of autoimmune diseases and inflammatory. The abnormal expression of T-box transcription factor (T-bet/TBX21)/trans-acting T cell specific transcription factor (GATA3)/retinoic acid receptor-related orphan receptor γt (RORγt)/forkhead/winged helix transcription factor (Foxp3), which has the transcription factor of Th1, Th2, Th17 and Treg, respectively, can reflect the subpopulation and status of CD4+ T subtypes [23]. By detecting the expression of transcription factor of each kind of cells through real-time PCR, we found that hydrogen protected mice from radiation-induced imbalance among these cells.

As cytokines secreted by T lymphocytes are the major substance in modulating the immune system, the cytokine profile of CD4+ T cells were compared among different groups [24]. It is well known that radiation could inhibit the proliferation of effective T cells by reducing the levels of Th1-type cytokines, while generating more Th2-type cytokines [25]. We found that the production of Th1-type cytokines (IFN-γ, IL-12) was enhanced in splenocytes from hydrogen-treated irradiated mice, whereas the levels of Th2-type cytokines (IL-4, IL-5 and IL-10) were decreased in H_2_ group. Both IFN-γ and IL-12 play important roles in promoting the differentiation of naïve T cells into Th1 cells and regulating immune reaction. IL-4, IL-5 and IL-10 play crucial roles in immunosuppression and mediating humoural immunity. Transforming growth factor-β1 induces the differentiation of Treg cells, whereas TGF-β1 in combination with IL-6 could result in the differentiation of Th17 cells [26,27]. It has been reported that whole-body irradiation increases Th17 and Treg cells [28]. In our study, we found that the level of TGF-β1 decreased significantly in H_2_ group. The levels of Th17-type cytokines (IL-17 and IL-23) were also reduced in H_2_ group. Besides the cytokines related to CD4+ T cells, we also found that the levels of other cytokines (TNF-α, IL-1b, GM-CSF) induced by the imbalance of CD4+ T cells decreased in H_2_ group. All those cytokines play a role in immune responses, inflammatory processes and haematopoiesis. On the basis of this, we suggest that the radioprotection effects of hydrogen on immune system might also be induced by regulating the imbalance of T cells.

The radiosensitivity of different immunocytes varies so much that the subpopulation of immune cells changes after certain doses of radiation, thus changed the percentage of T cells, as well as the immune status. In our view, hydrogen might act modulatory effects on immune functions by inhibiting radiosensitive cells from apoptosis after radiation exposure. Hydrogen protected immune cells from apoptosis through the scavenging of ROS, which was also determined in our study using a *in vivo* data.

Besides radioprotection effects, safety is another important requirement. To our knowledge, hydrogen can be produced by bacteria and circulate in the body, and the reaction between H_2_ and •OH produces water. It is physiologically safe for humans to ingest H_2_ at a relatively low concentration (<4%). Dissolving H_2_ in physiological saline has no risk of flammability or explosion and is easy to apply [9].

In summary, this study demonstrates that the radioprotective effect of hydrogen on immune system was attributed to its free radicals scavenging capacity. Hydrogen could relieve the damage caused by radiation to immune system through reducing apoptosis and autoimmune dysfunction *in vivo* through reducing the destroy mediating by toxic •OH. However, the possible mechanism and signalling pathway need to be studied in future.
